# The Promoting Effect of Animal Bioactive Proteins and Peptide Components on Wound Healing: A Review

**DOI:** 10.3390/ijms252312561

**Published:** 2024-11-22

**Authors:** Xiaoyu Fan, Jinhong Ye, Wanling Zhong, Huijuan Shen, Huahua Li, Zhuyuan Liu, Jie Bai, Shouying Du

**Affiliations:** College of Traditional Chinese Medicine, Beijing University of Chinese Medicine, Beijing 102488, China; 20230941505@bucm.edu.cn (X.F.); 20210941439@bucm.edu.cn (J.Y.); 20220941489@bucm.edu.cn (W.Z.); 20220941490@bucm.edu.cn (H.S.); 20230941516@bucm.edu.cn (H.L.); zhuyuan_liu@bucm.edu.cn (Z.L.)

**Keywords:** wound healing, protein, peptide, component, mechanism, formulation, dressing

## Abstract

The skin is the first line of defense to protect the host from external environmental damage. When the skin is damaged, the wound provides convenience for the invasion of external substances. The prolonged nonhealing of wounds can also lead to numerous subsequent complications, seriously affecting the quality of life of patients. To solve this problem, proteins and peptide components that promote wound healing have been discovered in animals, which can act on key pathways involved in wound healing, such as the PI3K/AKT, TGF-β, NF-κ B, and JAK/STAT pathways. So far, some formulations for topical drug delivery have been developed, including hydrogels, microneedles, and electrospinning nanofibers. In addition, some high-performance dressings have been utilized, which also have great potential in wound healing. Here, research progress on the promotion of wound healing by animal-derived proteins and peptide components is summarized, and future research directions are discussed.

## 1. Introduction

The skin, as the largest organ in the human body, is anatomically divided into three layers: the epidermis, dermis, and subcutaneous tissue [1]. It is the first line of defense to protect the host from external environmental damage and plays an important role in maintaining internal balance. Wounds open the door for foreign substances and organisms to enter the human body [2]. If wounds are not well cared for after injury, they can easily develop into chronic wounds (such as diabetic ulcers) [3], and large-scale loss of skin integrity may lead to serious disability or even life-threatening consequences [4]. However, skin damage is becoming increasingly common due to complications caused by burns, infections, scars, genetic diseases, and other diseases [5,6]. Approximately 2% of Americans suffer from chronic wounds, and the annual cost of wounds is as high as 28.1 billion to 96.8 billion dollars [7,8,9].

Skin wound healing refers to a series of dynamic and complex biological processes that restore normal structure and function [10]. The whole process involves four stages: blood clotting, inflammation, proliferation, and tissue remodeling [11]. It requires a variety of repair cells (keratinocytes, fibroblasts, endothelial cells, etc.), cytokines (epidermal growth factor (EGF), vascular endothelial growth factor (VEGF), granulocyte–macrophage colony-stimulating factor, etc.), and the extracellular matrix (ECM) [12]. Although wound healing is a spontaneous behavior of the body, the entire process is comprehensive and time consuming [13]. It may be affected by various factors, resulting in chronic wound healing retardation or delayed ulcer healing. Accelerating the wound healing process will significantly reduce the risk of wound infection [14]. This approach is not only beneficial for patients with severe skin damage caused by lacerations, burns, and accidental injuries but also has important value for the rapid recovery of postoperative patients [13]. Owing to the high morbidity and mortality rates associated with wounds worldwide, accompanied by high costs and low efficacy, wounds have caused severe economic and social damage.

Currently, treatment methods based on growth factors, cytokines, and cell therapy have been applied in the clinic [15]. However, these therapies still have significant shortcomings, such as the lack of long-term integration of cells, incomplete healing, and frequent occurrence of scar tissue. The high production cost makes protein drugs very expensive and difficult to afford. Moreover, they also have many disadvantages such as low stability, complex purification and expression processes, the burst release of wounds, immune rejection, and adverse side effects. At present, there is still a lack of effective wound treatments [15,16,17]. Moreover, with the increasing incidence and large economic burden of wounds, finding effective, economical, and practical drugs is urgently needed. Therefore, people have high expectations for developing drugs or effective treatment methods with potential for wound healing. With the goal of finding effective drugs, many researchers have shifted their attention to natural components [13,18,19], which hope to find natural products obtained from plants and animals as potential therapeutic drugs to accelerate wound healing [20,21].

At present, many active protein components (such as collagen peptides) obtained from animals have been shown to accelerate wound healing and prevent the formation of scars [3,22]. Because it comes from animal protein, it has the advantages of safety, reliability, and low price. Therefore, this article reviews the potential applications of animal proteins and peptide components in promoting wound healing from the perspectives of active components, sources, mechanisms, pathways of action, and related formulations Firstly, an overview of different categories of pro-healing proteins and peptides, animal sources, species, active ingredients, acquisition methods, and their effects on wound healing is shown in Table 1.

## 2. Components

### 2.1. Earthworm

Earthworms belong to the annelida phylum (in the animalia kingdom), are decomposers of ecosystems worldwide [60], and are among the earliest organisms on the phylogenetic tree. The coelomic fluid of earthworms is filled with 18 amino acids, fatty acids, microelements, lumbritin, lumbrofebrin, terrestrolum brolysin, purine, choline, cholesterin, and vitamins [61]. Many years ago, earthworms were used to treat various diseases [62]. The practice of extracting and using bioactive compounds from earthworms has widely circulated among indigenous peoples around the world, especially in Asia, including China, India, Myanmar, South Korea, and Vietnam [63]. As a Traditional Chinese Medicine, earthworms have significant medicinal value. The written records of the medicinal use of earthworms can be traced back to 1578 AD, and the medicinal methods of earthworms were recorded in detail in the Compendium of Materia Medica [64,65]. Previous studies have shown that earthworms have antiulcer [66], antioxidation [67], liver protection [68], anti-inflammatory [69], antibacterial [70], anticancer [71], antiapoptotic [72], anticoagulation, and fibrinolytic activities [73]. These properties and effects may contribute to the process of wound healing.

In recent years, with the continuous deepening of research on the ability of earthworms to promote wound healing, the study of their active ingredients has also been refined, from initial protein extracts to protein complexes with determined molecular weights. In addition to studying the whole body of earthworms, researchers have also studied the changes in bioactive components in the body of earthworms after amputation based on the characteristics of earthworm regeneration in order to further and comprehensively study the healing activity of earthworms.

A study of earthworm protein extracts revealed that the glycoprotein complex G90 [23] isolated from earthworm homogenate can improve the wound-healing activity of diabetic rats induced by alloxan, which may be due to increased proliferation of fibroblasts and epithelial cells. In addition, researchers have shown that G-90 has antioxidant, antibacterial and antimitogen activities, which are conducive to wound healing [74]. The biological activity of G-90 may be related to insulin-like growth factor, immunoglobulin-like growth factor [75], two serine peptidases encoding tyrosine [76,77] and EGF-like components [24].

The protein extract was further focused on extracts below 30 kd, and the extract EE [10] was studied through macroscopic observation and histopathological, blood, and immunohistochemical parameter determination methods. EE significantly shortened the wound healing time and reduced inflammation. The extraction method was further improved. After ultrafiltration, the precipitate was resuspended in phosphate-buffered saline and dialyzed to obtain an extraction solution, EE-1, with a protein concentration of 2.56 mg/mL [25]. EE-1 can promote deep second-degree burn wound healing, while the content of hydroxyproline in the skin reflects the collagen content [78], thereby reflecting the effect on the formation of scars [79]. These results show that, in addition to promoting wound healing, it can also accelerate the formation of collagen and ECM and reduce scar formation.

In addition to studying the whole body of earthworms, researchers have also studied the changes in bioactive components in the body of earthworms after amputation, based on the characteristics of regeneration after amputation. Earthworms can regenerate amputated parts of the body and stimulate the synthesis of EGF and VEGF. The effects of earthworms on promoting wound healing and reducing scar formation were significantly better than those of normal earthworms. The G-90′ [26] was extracted from the regenerated tissues of the earthworms after 3 days of tail amputation, after which three protein mixtures were isolated. ES2 promoted wound healing in vivo and in vitro. Its healing effect was significantly better than that of other protein mixtures. The functional proteins in ES2 were subsequently identified via the “bottom-up” proteomic analytic method. A total of 46 proteins were identified. Through cross-identification via LC–MS/MS and transcriptome analyses, 23 functional proteins were obtained. The better healing-promoting activity of earthworms after amputation may be related to the upregulation of the heat shock protein HSP70 and lysozyme genes, suggesting that the heat shock protein HSP70 and lysozyme are functional proteins in earthworms that promote wound healing [27]. A new collagen-like peptide, col4a1, from amputated earthworms was subsequently screened and identified via high-throughput techniques, including transcriptomics, proteomics, and mass spectrometry [13]. Col4a1 was cloned and expressed, and its effects on wound healing and mechanism were comprehensively explored. The results showed that it had a significant effect on wound healing both in vivo and in vitro. Col4a1 effectively enhanced the viability, proliferation, and migration of fibroblasts, the formation of granulation tissue, and the deposition of collagen.

At present, research on the active ingredients that promote wound healing in earthworms has focused mostly on mixtures. The active ingredient is a mixture of proteins and peptides. The simple extraction method leads to a complex composition of the active ingredients obtained. There are currently no reports on specific components, which may be related to the difficulty of further extraction, separation, and purification of proteins, as well as the poor activity of biosynthetic proteins. The inability to determine the specific ingredients presents certain difficulties for further pharmacological and formulation research, so further research on active ingredients is still needed.

### 2.2. Periplaneta

Periplaneta is one of the most famous health-related insects. It has strong vitality and reproductive ability and is widely distributed in subtropical and tropical regions around the world. *Periplaneta americana* (PA) is a species of Periplaneta, and an important Traditional Chinese Medicine that promotes blood circulation, eliminates purulent effects, and regulates immunity. Its medicinal components include mainly peptides, proteins, and polyols [80].

Physiological and pharmacological studies have shown that the components of PA have good tissue repair ability. A Traditional Chinese Medicine formulation called Kangfuxin (KFX) was developed from ethanol extracts from the dried body of PA and has been applied in the clinic. It is used to treat various types of injuries, such as burns, scalds, knife wounds, and other types of trauma and refractory ulcers, with significant clinical effects and wide applications.

The ethanol extract of PA (PAL) has been proven to promote wound healing [31,81]. As a marker of vascular endothelial cells, CD31 was specifically expressed [82]. Immunohistochemical results revealed CD31-specific staining was observed in the low- and medium-dose PAL groups. The collagen in each PAL dose group was relatively thick, which clearly distinguished the bundled collagen, and the regeneration and recombination ability of the collagen was better. The results showed that PAL had a certain anti-scar effect [83].

Further research was subsequently conducted on the two proteoglycan complexes, PaPPc2 and PaPPc3, obtained from PA [32,84]. The molecular conformation of PaPPc2 was dominated by the β-strand conformation, whereas that of PaPPc3 was dominated by random coils. PaPPc2 and PaPPc3 can be connected to polysaccharides through O-glycosidic bonds. Further research was subsequently conducted on the pharmacological effects of PaPPc2 and PaPPc3, both of which can promote endothelial cell differentiation, angiogenesis, and collagen synthesis and thus promote wound healing.

At present, although there are drugs with active extracts of PA in clinical application, relatively few studies have investigated their active ingredients. These studies have also focused mostly on the active extract. The products obtained through ethanol extraction [19,31,32] have not been further separated and purified, which also has problems such as complex components and unclear specific active ingredients that take effect.

### 2.3. Amphibians

Amphibians live in harsh and complex environments, requiring them to adapt to both marine and terrestrial environments. Exposed skin makes them vulnerable to microbes, ultraviolet radiation, and other types of injury [85]. Through long-term natural selection, amphibians have evolved unique and efficient skin peptide defense systems, mainly including antimicrobial peptides, antioxidant peptides, lectins, and protease inhibitors, which can play roles in antibacterial, antioxidant, and immune regulation [86,87,88,89,90,91,92,93,94]. Previous studies have shown that the skin of amphibians can repair itself quickly after injury [95,96]. Therefore, researchers have hypothesized and confirmed the existence of the necessary material basis for promoting wound healing in the skin [96,97,98]. There are various types of amphibians, and it has been proven that the skin secretions of various amphibians have strong healing-promoting activity.

Frogs are a species of amphibians whose ability to secrete active ingredients that promote wound healing has been extensively studied. Odorous frogs can secrete various bioactive peptides [90]. Several bioactive peptides [43,44] extracted from *Odorrana Margaretae* promoted wound healing in HaCaT cells and HSF cells in a time-dependent and dose-dependent manner and promoted wound healing in a mouse full-thickness skin wound model. The bioactive peptides extracted from *Odorrana andersoni* [39] also promoted wound healing in HaCaT and HSF cells in a time-dependent and dose-dependent manner. Moreover, it promoted wound healing in a dose- and time-dependent manner in a mouse full-thickness skin wound model while preventing the formation of chronic wounds and scars. Compared with existing small molecule compounds and growth-factor-based wound-healing drugs, it has obvious advantages. Extracting new short peptides from *Pelophylax nigromaculatus* can promote the migration of HSF cells and accelerate scratch healing in a time-dependent manner [47]. A new peptide was identified from the skin of *Nanorana ventripunctata* [36], which exhibited strong wound healing-promoting activity in a mouse dermal wound model. It can directly enhance the formation of glial cells, accelerate re-epithelialization at the wound site, and promote the formation of granulation tissue.

In addition to frogs, other amphibians still contain peptides that promote wound healing. TK-CATH was identified from *Tylototriton kweichowensis* [52]. It can effectively induce the production of a variety of cytokines, chemokines, and growth factors related to wound-healing activity in a mouse full-thickness wound model and accelerate skin wound healing. In addition, it also has strong anti-inflammatory activity. The results suggested that it may influence the inflammatory phase and the new tissue formation phase during wound healing [52].

In recent years, many reports have shown that peptides identified from amphibians significantly affect wound healing, as shown in Table 2. A summary analysis of these peptides reveals that most of them are polypeptides, with only two oligopeptides. The number of amino acids is concentrated between 10 and 30, but no similarities have been found in the amino acid composition. In terms of structural modification, seven peptides contain disulfide bonds in their structures, with fewer N-terminal and C-terminal modifications. Further research is needed on the relationship between its structure and bioactivity.

At present, most studies on amphibian extracts focus on peptides, and the research ideas are relatively systematic and complete. The research approach for peptides in these amphibians can be summarized as extraction, isolation, identification, synthesis, pharmacological, and mechanistic studies. Among them, peptides are separated mainly by gel permeation chromatography, reversed phase high-performance liquid chromatography RP-HPLC, and other methods. The structure of the peptide was subsequently identified via Edman degradation, mass spectrometry, Ladder sequencing, or cDNA sequencing. Finally, the target peptide was commercially synthesized through solid-phase synthesis and other methods. Next, its activity and mechanism were studied. The existing research directions focus mostly on antibacterial, anti-inflammatory, and antioxidant aspects. Although it has been proven to promote wound healing, its mechanism is still related to antibacterial, anti-inflammatory, or antioxidant effects. Its healing-promoting effect is mostly achieved by improving the microbial environment of the wound.

### 2.4. Marine Organisms

Marine organisms, such as fish, jellyfish, sponges, and other invertebrates, contain large amounts of collagen. As a significant source of collagen, marine organisms have greater advantages than other sources do, such as being metabolically compatible, being able to be utilized free from religious constraints, and being free of animal pathogens [101,102,103]. Moreover, marine organisms have abundant collagen resources and low prices, which are widely found in more than 20 million tons of byproducts, such as fish skin, heads, fins, and viscera [104]. Collagen, an important structural protein in the animal ECM and connective tissue, plays an important role in supporting the formation, tensile strength, and flexibility of joints [105,106,107,108]. Marine collagen can also reduce wrinkles, improve skin elasticity, and enhance the overall structure and appearance of the skin.

Active ingredients that promote wound healing have been found in various marine organisms, including *Tilapia Piscidin* [18], sea cucumber [53,54,109], *Sipunculus nudus* [3], *Salmon sala* [55], *Nibea japonica* [56], and *Theragra chalcograma* [57]. These ingredients have all been proven to promote cell migration and proliferation and have shown good performance in vivo. They can accelerate wound healing in mice, can even shorten scar formation and clearance times, and have a scar-free healing effect. They have good biological activity in promoting wound healing and inhibiting scar formation.

Some researchers have directly validated the effectiveness of fish skin collagen and its promoting effect on patients with venous leg ulcers (VLUs) through clinical trials [110]. Compared with the placebo group, the fish in the collagen gel group exhibited greater ulcer healing after administration and significantly improved the quality of life of patients. These findings indicate that fish collagen can indeed improve the condition of VLUs in patients and improve their quality of life.

There are rich and diverse marine species, and marine resources are also enormous. The ingredients that promote wound healing are also diverse. Research on the bioactive components of marine organisms that can promote wound healing has been continuously conducted, and many bioactive components of marine organisms have been proven to have significant effects on promoting wound healing. Marine collagen has been shown to promote skin re-epithelialization, vascularization, fibroblast migration, and healing ability. It even has the effects of increasing skin elasticity, reducing wrinkles, and preventing aging [111]. In recent years, promoting wound healing with natural products has become a trend. Although the healing-promoting active components from marine organisms have been shown to be fully applicable, they are still underutilized natural resources [112]. Therefore, future research on promoting wound healing in marine organisms should focus more on the full utilization of natural resources, in addition to bioactive components.

### 2.5. Scorpions

Scorpions are ancient chelators with a wide range of habitats [113]. Scorpion venom is a complex and abundant source of active substances. It is an important weapon used by scorpions to defend themselves, deter opponents, and catch prey [114]. Its abundant bioactive substances have also received widespread attention. Two scorpion venom peptides extracted from scorpion venom effectively shorten the wound healing time and have strong anti-inflammatory and antibacterial effects [58,59]. The good healing ability of these peptides is closely related to their antioxidant and antibacterial abilities.

According to the literature, studies on the activity of scorpion venom have focused mostly on insecticidal, antibacterial, antifungal, antiviral, antiparasitic, and antioxidant effects [115,116,117,118,119]. Few studies have investigated the promotion of wound healing [58,59]. However, these reports further improve the research on scorpion venom, suggesting that more attention can be paid to the wound-healing-promoting components in scorpion venom. It is interesting to note that the existing types of animals that have been found to contain protein or peptide components that promote wound healing have smaller body types and a weaker ability to cope with environmental changes. Perhaps this is also why they can secrete various protective ingredients, such as those that can be used to promote wound healing, to cope with harsh and complex living environments, repair damaged wounds, and maintain the stability of their internal environment.

In summary, the ingredients that promote wound healing in earthworms, PA, and marine organisms are mostly concentrated in the extract mixture, and their bioactive ingredients are mainly glycoprotein complexes or collagen. There is relatively few research on single protein components. The components of glycoprotein mixtures are complex and difficult to clarify. Collagen, the major protein in ECM [120,121], is involved mainly in the development and migration of cells and tissues [111,122]. In addition, it significantly improves the wound-healing process by stimulating and promoting the production of keratinocytes and fibroblasts near the wound [122]. Summarizing the existing research on protein monomer components that promote wound healing, only PvESII has been reported to have an accurate glycoprotein structure, with a unique structure such as the EGF domain [28], which is a growth factor analog that can bind to the epidermal growth factor receptor (EGFR). EGF is widely present in human and animal tissues and can promote or inhibit the growth of various cells. Epidermal cells, fibroblasts, and endothelial cells are all targeting cells of EGF. In acute trauma, EGF promotes the proliferation and migration of keratinocytes by regulating the expression of keratin 6 and keratin 16 in the keratinocyte proliferation signaling pathway [123]. It can promote wound regeneration and epithelialization and improve the tensile strength and tension of the wound. Owing to the presence of EGF-like domains, PvE-3 can also bind to EGFR, initiate wound-healing signals, and promote wound regeneration and epithelialization, and this mechanism of action is like that of current clinical treatment methods based on growth factors. There are also reports that proteins such as HSP70 and lysozyme promote healing, both of which are related to the endocytosis pathway and the process of endocytosis. However, their specific structure and active sites have not been elucidated [27].

The current lack of research on monomeric protein components may be due to the limited methods used to study protein components, the low separation efficiency, and the high cost. With the further development of biotechnology and in-depth research on the structure and function of various proteins, separation and purification technology for proteins has also rapidly developed. Commonly used techniques such as salting-out, isoelectric precipitation, organic solvent precipitation, dialysis, and ultrafiltration can separate proteins on a large scale. However, these technologies have the drawbacks of low resolution and high impurity content, requiring subsequent dialysis or ultrafiltration to remove salts or organic solvents. Chromatography and electrophoresis are high-precision separation methods that can further purify proteins, but they are difficult to use for large-scale separation because of limited sample processing. Therefore, it is difficult to obtain sufficient protein components that can be used for activity research. In addition to common separation and purification, biofermentation engineering or protein recombination technology can also be used to prepare proteins, but there are also different problems. Owing to the complexity of protein conformation and different conformations of carbohydrate chain modifications, most proteins produced by biofermentation have poor or no biological activity. Moreover, protein recombination technology has high costs and is difficult to promote further. Owing to limitations in protein separation and purification technology, it is difficult to obtain bioactive proteins from monomers. Therefore, current research on the ability of earthworms, PA, and marine organisms to promote wound healing has focused mostly on extracting mixtures and has not delved into monomer components.

The components that promote wound healing in amphibians and scorpions are mostly peptides with known amino acid sequences. The number of amino acids in these peptide segments ranges from 5 to 81, with the majority being less than 40. Two peptides have C-terminal modifications linked to -NH_2_. A peptide links -Pyr at the N-terminus. Seven peptides contain disulfide bonds, but no correlation between their structural characteristics and biological activity has yet been found. Compared with protein components, peptides have a simpler conformation, known amino acid sequence, smaller molecular weight, and lower production cost, which makes them potentially widely used in clinical practice. Moreover, the small molecular weight of peptides is more suitable for preparing skin wound formulations for topical administration through biological barriers. As external drugs, small-molecule drugs are more likely to reach higher concentrations in superficial tissues [123]. Moreover, peptides are more likely to maintain their natural structure through N-terminal acetylation or C-terminal amination, increasing their resistance to external enzymes [123]. In addition, cysteine residues can be cyclized by disulfide bonds, improving the stability of the peptide.

## 3. Mechanism

**Figure 1:**
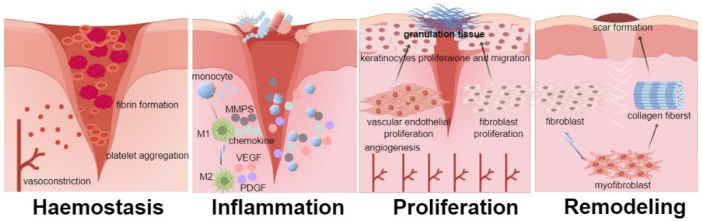
Schematic diagram of each stage of wound healing. Hemostasis phase: vasoconstriction, platelet aggregation, and fibrin formation. Inflammatory phase: monocytes migrate to the wound and differentiate into M1 macrophages. M1 macrophages secrete inflammatory mediators and transform into M2 macrophages. M2 macrophages secrete growth factors. Proliferation phase: angiogenesis, fibroblast proliferation, myofibroblast differentiation, and collagen deposition. Remodeling period: fibroblasts differentiate into myofibroblasts, producing type I and III collagen. In the later stage, MMPs degrade type III collagen, and type I collagen forms scars.

Skin wound healing involves a series of complex and continuous physiological processes, which are divided into four stages, blood clotting, inflammation, proliferation, and tissue remodeling [11], as shown in Figure 1. This process involves the activation of platelets, neutrophils, macrophages, endothelial cells, keratinocytes, and fibroblasts, as well as the secretion of growth factors, inflammatory factors, chemokines, and other substances [124]. Immediately after skin injury, hemostasis begins, including the vasoconstriction phase, thrombus formation phase, and blood clotting phase, which is the body’s first response to the wound to prevent bleeding and pathogen invasion. The characteristics of this process are vasoconstriction, platelet aggregation, and fibrin formation [125]. The inflammatory phase is subsequently activated by platelet aggregation and the release of inflammatory mediators. During the inflammatory phase, damaged cells guide neutrophils to enter the wound and kill pathogens by increasing damage-associated molecular patterns or by using signals such as hydrogen oxide and chemokines to form gradients [125]. Monocytes migrate to the wound, differentiate into macrophages, and begin to polarize. M1 macrophages promote inflammation, degrade and clear damaged tissue, and further stimulate neovascularization by secreting various chemokines, matrix metalloproteinases (MMPs), and other inflammatory mediators. M2 macrophages can promote the secretion of various growth factors, including VEGF and platelet-derived growth factor [126]. Macrophages also produce important signaling molecules, which subsequently activate keratinocytes, fibroblasts, and endothelial cells [127]. In the late stage of inflammation, T lymphocytes stop interferons-γ to regulate the inflammatory response and allow the wound to progress to the proliferative phase [128,129]. The proliferation phase is characterized by angiogenesis, fibroblast proliferation, myofibroblast differentiation, and collagen deposition. M1 macrophages transform into the M2 subtype and promote proliferation by secreting anti-inflammatory cytokines and collagen precursors, thereby stimulating fibroblast proliferation [130]. Fibroblasts and endothelial cells at the wound begin to proliferate and form ECM-filled granulation tissue, generating blood vessels and a new matrix to fill tissue defects. Keratinocytes proliferate and migrate to wounds, where they initiate epithelialization [30,131,132]. The proliferation phase mainly protects the wound by stimulating the formation of granulation tissue. The final stage of wound healing involves ECM healing and remodeling [133]. During the tissue remodeling phase, collagen fibers and excessive ECM are degraded [131]. This stage begins 2–3 weeks after the formation of the wound or within 1 year of wound formation. In the process of tissue remodeling, TGF-β1 signaling stimulates fibroblasts to differentiate into myofibroblasts, thereby producing type I and type III collagen. Collagen fibers are enhanced through cross-linking under the catalysis of transglutaminases and lysyl oxides. In the later stages of tissue remodeling, MMPs degrade type III collagen, leading to scar formation accompanied by abundant type I collagen [134]. In addition, in chronic wounds, bacteria often colonize microbial membranes to evade the host immune response and the effects of antibiotics. It can also reflexively induce the expression of inflammatory cytokines. Proteinase and reactive oxygen species (ROS) increase, and the degradation of the ECM and growth factors by proteases and ROS delays wound closure. Multiple cells and signaling pathways are involved in the complex process of skin wound healing. To summarize the mechanism of action of existing animal proteins and peptide active ingredients on wound healing, they act on different stages of wound healing through different pathways. 

### 3.1. The TGF-Beta Pathway

The TGF-β signaling pathway is a complex, multifunctional pathway that regulates a variety of cellular processes, such as proliferation, differentiation, migration, apoptosis, and ECM synthesis [135]. The TGF-β signaling pathway is activated by cytokines, growth factors, or mechanical stress, which bind to TGF-β RII and then phosphorylate and activate TGF-β RI. TGF-β RI further activates Smad2/3 and interacts with Smad4. The Smad complex translocates to the nucleus and regulates the transcription of the integrin, E-cadherin, c-Myc, and IL-6 genes [136]. In the TGF-β family, TGF-β1 plays a dominant role in skin wound healing, mainly by mediating pathological healing, such as chronic inflammation and excessive scar formation [137]. During the inflammatory phase, platelets and white blood cells secrete TGF-β as a chemotactic factor, promoting fibroblast proliferation and migration to the wound site [138,139]. During the proliferation phase, TGF-β1 promotes the migration of keratinocytes, angiogenesis, and granulation tissue formation [138]. During the remodeling phase, TGF-β1 stimulates the differentiation of fibroblasts into myofibroblasts, promoting wound contraction and collagen production [140].

SNCP [3], obtained from marine organisms could downregulate the expression levels of TNF-α, IL-1β, TGF-β1, and TGF-β RII and upregulate the expression level of Smad7. TNF-α and IL-1β are important inflammatory factors and indicators of inflammatory response. Inflammation is necessary to resist the invasion of foreign pathogens and clear fragments of tissue, but excessive inflammation can cause damage to normal tissues and lead to fibrotic diseases [141]. Excessive inflammation during wound healing can lead to delayed healing and scar formation [142]. SNCP effectively shortens the inflammatory phase and has anti-inflammatory effects by significantly reducing the expression levels of TNF-α and IL-1β. In addition, TNF-α and IL-1β could dose-dependently reduce collagen synthesis. TNF-α could strongly promote the secretion of MMPs, degrade collagen, and block the mRNA transcription of type I/III pro-collagen [143]. TGF-β1 is an important driving factor in fibrosis and is associated with abnormal scar hyperplasia [144]. Downregulation of TGF-β1 and TNF-α by SNCP could effectively prevent scar hyperplasia. Smad7 is a negative-feedback-regulated inhibitory protein that strongly binds to TGF-βrI, inhibits Smad2/Smad3 phosphorylation, and blocks the TGF-β/Smads signaling pathway [145,146,147]. The TGF-β/Smads signaling pathway is an important pathway that affects scar formation and extracellular matrix deposition after trauma [148]. The significant upregulation of Smad7 expression level inhibits and blocks the TGF-β/Smads signaling pathway. This is also the reason SNCP regulates collagen formation and remodeling and inhibits scar hyperplasia. Therefore, SNCP mainly accelerates the process of wound healing by anti-inflammatory activity and regulates collagen formation and remodeling, affecting the inflammatory, proliferation, and remodeling phases.

The peptide AH-90 [41] obtained from amphibians could promote the expression of TGF-β1, phosphorylated Smad3, and α-SMA, but does not cause upregulation of pro-inflammatory factors such as IL-6 and TNF-α. The results showed that AH-90 could dose-dependently upregulate the expression of TGF-β1. TGF-β1 upregulates the expression of α-SMA by phosphorylating Smad3 [149]. The expression of α-SMA is an important characteristic of fibroblast differentiation into myofibroblasts [150]. The transformation from fibroblasts to myofibroblasts is also an important step in skin healing [151]. Therefore, AH-90 further upregulated the levels of phosphorylated Smad3 and α-SMA by promoting the expression of TGF-β1. Then, it activated the TGF-β pathway to promote the proliferation of myofibroblasts, accelerated the process of wound re epithelialization in mice, and caused faster contraction of granulation tissue compared to the control group. The histological section also confirmed this phenomenon. The expression levels of IL-6 and TNF-α were not affected, indicating that AH-90 activated the TGF-β signaling pathway and promoted wound re-epithelialization and granulation tissue contraction by TGF-β1 but did not cause prolonged activation of the inflammatory phase. On the contrary, the control group was still in the inflammatory phase at this time, and there was still thick granulation tissue on the wound site in the mice.

### 3.2. The PI3K/AKT Pathway

The PI3K/AKT pathway is one of the most important pathways in wound healing and includes the MAPK signaling pathway, the VEGF signaling pathway, the mTOR signaling pathway, etc. This pathway is closely related to the formation of the epidermal barrier, collagen synthesis, and angiogenesis [152,153]. It is also an important factor involved in the process of acute wound healing and maintaining tissue homeostasis [154]. AKT is a serine/threonine kinase that is an important signaling center for various cellular functions. PI3K-dependent AKT activation further affects the activity of downstream pathways, such as proliferation, angiogenesis, aging, regulation, and cell survival [155,156]. The PI3K/AKT pathway is initiated by the binding of cytokines and growth factors to receptor tyrosine kinases (RTKs) [157]. RTKs activate PI3K and further activate AKT. AKT phosphorylation leads to the activation of mTOR C1, which in turn promotes the translation of several RNA transcripts that promote migration and proliferation, regulate the cell cycle, and affect the process of wound healing. During the process of wound healing, the PI3K/AKT signal is involved in mainly the inflammatory and proliferative phases, which can promote epithelial to mesenchymal transition, cell proliferation, and angiogenesis and reduce the inflammatory response [158,159,160,161]. At present, some animal proteins and peptide components have been proven to be related to the PI3K/AKT pathway in promoting wound healing.

The collagen-like peptide col4a1 [13] obtained from earthworms could bind with integrin α2β1 to activate downstream RAS/MAPK signaling pathways, significantly increasing the expression of collagen I and III and elastin at the wound site. Collagen, as the main protein in ECM, has been shown to accelerate chronic wound healing [120,121]. In most cases, the role of collagen in the healing process is to attract fibroblasts and other cells, providing a natural scaffold or matrix for newly formed tissue [121,162]. The centripetal displacement at the edge of the wound serves as a marker of the healing and proliferation stage, requiring activation and migration of fibroblasts and a large amount of deposited ECM to the damaged tissue [163]. The mice in the col4a1 treatment group showed significantly accelerated wound healing and fibroblast recruitment, which was consistent with the high expression of collagen I and III at the wound site, indicating that col4a1 promotes healing by acting on the proliferative and remodeling phases of wound healing.

In addition, besides earthworms, Bv8-AJ [33] was a peptide identified from amphibian secretions, which also promoted cell proliferation and wound healing through the MAPK signaling pathway. Bv8-AJ could activate the phosphorylation of ERK, JNK, and p38 in KC cells in a dose-dependent manner, thereby activating the MAPK pathway and promoting the secretion of IL-1α and IL-1β. The IL-1 signaling pathway is a key pathway for controlling the proliferation of keratinocytes and fibroblasts. IL-1 activates the expression of mitotic factors by activating the peroxisome proliferator-activated receptor β/δ in fibroblasts [164], thereby directly promoting the proliferation of keratinocytes and fibroblasts and ultimately accelerating the beginning and end of the inflammatory phase. This result was consistent with the histopathological section results. Compared with the control group, the Bv8-AJ group showed earlier infiltration of inflammatory cells and earlier reduction in inflammatory cell infiltration. This indicated that Bv8-AJ promotes wound healing by shortening the inflammatory phase.

### 3.3. The NF-Kappa B Signaling Pathway

The NF-kappa B signaling pathway is a typical pro-inflammatory signaling pathway. During the wound healing process, it mainly acts on the inflammatory phase through its anti-inflammatory and antioxidant effects, improving the inflammatory microenvironment of the wound or promoting wound healing through the regulation of the immune system [165]. NF-kappa B, an inducible transcription factor, is present in all types of nucleated cells [166]. It regulates the expression of MMPs by activating innate immune responses, cell proliferation, and cell migration. The promotion of wound healing promotes the secretion and stability of cytokines, chemokines, adhesion molecules, growth factors, and pro- or antiapoptotic proteins [165,167,168]. The NF-kappa B signaling pathway functions through two main signaling pathways. The typical (classical) pathway is activated by stimuli such as cytokine receptors, tumor necrosis factor receptors (TNFRs), pattern recognition receptors, T-cell receptors, and B-cell receptors [169]. The atypical (alternative) pathway is activated by a subset of the TNFR superfamily [170]. Among these pathways, the typical pathway is often closely related to wound injury.

SCPs [55] are collagen peptides isolated from marine organisms, which promote the NF-κB signaling pathway by upregulating the secretion of NOD12 and BD14, thereby promoting wound healing. The results of a previous study showed that SCPs could significantly promote NOD2 secretion. NOD2 is closely related to cutaneous microbial colonization, and cutaneous microbial colonization is believed to rigorously regulate the innate immune response of the wound-healing process [164,171,172]. The NOD2/NF-κB pathway plays a crucial role in innate immune regulation [173]. Compared with the control group, the SCPs group showed an increase in NOD2 content at the wound site and a significant change in the wound microbiota. The changes in microbiota further lead to changes in wound immune response. NOD2 undergoes a cascade reaction upon exposure to harmful bacterial stimulus, inducing activation of the NF-κB pathway, immune system, and antibacterial response, such as the release of antibacterial peptide (AMP) [56,174]. After treatment with SCPs, one of the classic members of the AMP family, BD14, significantly increased. BD14 can further regulate the microbial community of wounds [172]. The changes in wound microbiota caused alterations in the levels of inflammatory factors between the SCPs-treated group and the control group, altering the process of wound healing. Ultimately, SCPs upregulate NOD12 to regulate the NF-κB pathway and promote BD14 secretion, thereby controlling the inflammatory phase, increasing angiogenesis and collagen deposition, and promoting wound healing.

### 3.4. The JAK/STAT Signaling Pathway

The JAK/STAT signaling pathway is a key pathway that mediates cellular responses to various cytokines and growth factors (such as interleukins, interferons, and colony stimulating factors). The JAK/STAT pathway is involved in regulating various aspects of wound healing, such as inflammation, angiogenesis, ECM formation, and cell proliferation and migration [175]. After the binding of cytokines and growth factors to their specific receptors, the phosphorylation of JAKs and STATs activates the JAK/STAT signaling pathway. Phosphorylated STATs then dimerize and are translocated to the nucleus, where they regulate the transcription of target genes [176]. However, this pathway requires strong cellular control, and its loss of control can lead to chronic inflammation [177]. Therefore, in the healing of chronic wounds, normal regulation of the JAK/STAT pathway is particularly important, as it is one of the main signaling pathways involved in the use of growth factors and cytokines [178].

The extract PAE [19] from cockroaches could significantly upregulate the expression levels of p-JAK1, p-JAK2, and their downstream molecule p-STAT3 in both in vitro and in vivo experiments. p-Smad3 was also significantly increased, which promoted healing by activating the JAK/STAT3 signaling pathway. Compared with the control group, the PAE group showed significant fibroblast proliferation and fibrosis, leading to the formation of scar tissue. Histopathological analysis revealed an increase in epithelial repair, follicle regeneration, and additional fibrous tissues in the PAE-treated groups. It showed a significant increase in the wound healing rate compared to the control group.

**Figure 2:**
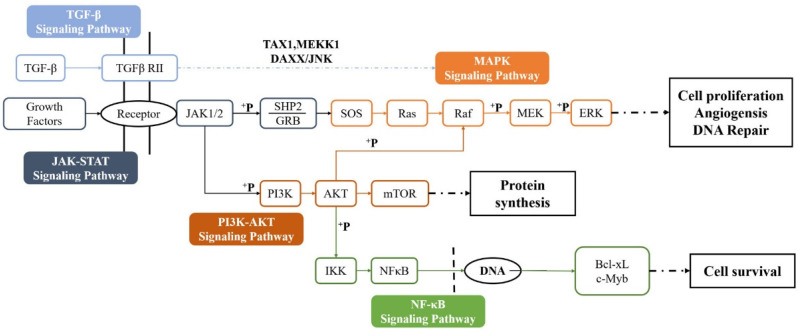
Crosstalk relationships between signaling pathways. The TGF-β signaling pathway is activated by cytokines, growth factors, or mechanical stress, regulating various cellular processes such as proliferation, differentiation, migration, apoptosis, and ECM synthesis. The PI3K/AKT pathway is initiated by the binding of cytokines and growth factors to RTKs and is related to the formation of the epidermal barrier, collagen synthesis, and angiogenesis. The typical pathway of the NF-κB signaling pathway closely related to wounds is activated by stimuli such as cytokine receptors, TNFR, PRRs, TCR, and BCR, which improve the inflammatory microenvironment of wounds and regulate the immune system through their anti-inflammatory and antioxidant effects. The JAK/STAT signaling pathway is activated by the binding of cytokines and growth factors to their specific receptors, the phosphorylation of JAKs and STATs, and is involved in regulating inflammation, angiogenesis, cell proliferation and migration, and ECM formation. The four key pathways interact with each other and work together at various stages of wound healing.

Although different components act on different targets at different stages of wound healing, they mainly focus on these four pathways. The different pathways through which animal proteins and peptides promote wound healing in existing reports is summarized in Table 3. There is a crosstalk interrelationship between these four pathways, as shown in Figure 2.

Summarizing the existing research, it could be found that the mechanism of animal protein and peptide components was mostly focused on the PI3K/AKT pathway, among which MAPK was currently the most studied pathway in wound healing. This pathway is one of the most important pathways in the wound-healing process, closely related to the formation of the epidermal barrier, collagen synthesis, and angiogenesis [152,153]. It is an important factor involved in acute wound healing and maintaining tissue homeostasis [154]. In future research, it is suggested to focus on the effects of bioactive proteins or peptides on the PI3K/AKT pathway. In addition, related upstream and downstream pathways can be studied to comprehensively elucidate the healing activity and mechanism of the components.

## 4. Formulation and Dressing 

Owing to their instability, proteins and peptide components are highly susceptible to inactivation by pH or various enzymes during systemic administration. When administered orally, they often face the influence of gastrointestinal breakdown or first-pass effects, and when administered by injection, they may be inactivated, owing to their susceptibility to various enzymes and receptors in the blood. Therefore, when protein and peptide components are used to treat skin injuries, topical administration can effectively avoid the problem of protein inactivation, making topical administration a more efficient and appropriate method. Compared with systemic administration, topical administration has significant advantages, as it can directly target the affected area, minimize systemic drug exposure, and, consequently, reduce systemic side effects. At the same time, it can also avoid the liver first-pass effect [179,180,181]. Topical administration can also achieve painless administration and improve compliance for patients with swallowing difficulties, making it more convenient. However, topical administration also faces other challenges, such as the need for repeated administration due to the short stay time at the affected site, the possibility of patient allergies caused by dressings, poor biocompatibility, skin barrier damage leading to transdermal water loss and increased inflammation, susceptibility to secondary infections, fast degradation rate, and poor stability [182,183,184,185,186,187]. Therefore, to solve such problems, various safe and effective formulations have been gradually studied, attempting to improve the wound microenvironment, increase drug retention time, and block microbial invasion.

### 4.1. Hydrogel

Gel is the most common formulation of animal protein and peptide components used to promote wound healing. It has received increasing attention as a wound dressing. As physical and chemical crosslinking agents with a 3D polymer material network, hydrogels can provide an ideal environment for wound healing.

Hydrogels can provide a moist environment at the wound site, helping to absorb the wound exudate without autolysis. Moreover, they have a high water-holding capacity, do not penetrate bacteria, prevent a large amount of exudation from accumulating on the wound, and provide a suitable growth environment for tissue regeneration. They also allow for gas exchange and can carry and control the release of bioactive molecules. Hydrogels have potential advantages in acute and chronic wound healing [188,189,190,191]. Hydrogels can be prepared by the physical and chemical crosslinking of nanoparticles, nanofibers, and other bioactive compound mixtures in a precursor solution [192]. Active ingredients can be directly loaded on hydrogel materials [193] or loaded on hydrogel materials in the form of nanocrystals [194], microspheres [195], nanoparticles [196], or nanosheets [197]. Moreover, gel materials can be modified accordingly to achieve temperature, pH, light, and other targeting effects. Hydrogels are good drug delivery carriers.

Chen [198] et al. blended polyvinyl alcohol, hydroxypropyl chitosan, and carbomer as materials and glycerin as a plasticizer to embed PA extract in a hydrogel film (PAE/film) via the solution cast method. The prepared PAE/film significantly accelerated the wound healing process in both full-thickness skin defects and scale wound model mice. During the entire experiment, compared with those of the other treatment groups, the wound area was significantly reduced, and the re-epithelialization rate was significantly increased, demonstrating a superior ability to promote wound healing. Its ability to promote healing is closely related to the superior water absorption and permeability of the hydrogel materials. It can effectively absorb wound exudate without autolysis. The high water-holding capacity of this material prevents the accumulation of a large amount of exudate on the wound bed, providing a suitable growth environment for tissue regeneration.

In addition, as physical and chemical crosslinkers with three-dimensional network structures, hydrogels can carry different peptides at the same time to play a synergistic role. Because wounds easily cause bacterial colonization and the formation of microbial membranes to delay wound healing, they can also promote wound healing through the formulation of hydrogels combined with active peptides and antibacterial peptides. Feng [199] et al. extracted collagen from marine fish scales and mixed it with sodium alginate, polymyxin B sulfate, and antimicrobial peptides to prepare a hydrogel dressing. The prepared hydrogel can effectively inhibit Escherichia coli and Staphylococcus aureus, accelerate reepithelization, collagen deposition, and angiogenesis to promote full-layer wound healing in a rat model and effectively ameliorate the bacterial infection problem that easily occurs in skin wounds.

### 4.2. Microneedles

Microneedles (MNs) are a new type of physical penetration enhancement technology that can directly bypass the stratum corneum and enter the epidermis/dermis to deliver drugs without contacting blood vessels or painful neurons. It has many advantages, such as no pain, minimal invasiveness, simple production, and convenient administration [200,201,202]. It has unique advantages in wound healing and tissue regeneration. The tips of MNs can easily pass through the physical barrier of the wound site, such as blood clots, scars and exudates, and continuously release the drug, which can greatly improve the efficacy of drugs [203]. In addition, it also provides natural mechanical stimulation for tissue regeneration during wound healing. Through tissue penetration and mechanical stimulation, collagen deposition and reorganization are induced [203,204]. MN arrays can support the insertion area and change the local stress environment [205]. These effects are beneficial for reducing scar formation during wound healing. MNs allow for the loading of a wide range of drugs [206,207] and can overcome the drug resistance of biofilms in the wound bed [208]. MNs have developed into various forms, such as solid MNs, coated MNs, hollow MNs, dissolvable MNs, and swelling MNs [209]. Drugs can be loaded directly into the internal channels of hollow MNs in liquid [210], can be made into nanocrystals [211], or can be directly encapsulated in microspheres [212] and polymer micelles [213] and then loaded onto MNs. Alternatively, drugs can be loaded onto nanoparticles and added to MN patches [214]. Nanoparticles can also be used as reinforcing agents and cross-linking centers to make hydrogel MN patches, further enhancing the targeting of the preparation [215]. As a formulation method, MNs can achieve the goals of targeting, sustained release, and prolonging the residence time of the affected area through various forms of formulation. It is a good carrier for healing drug formulations.

Yu [216] et al. established a multifunctional MN patch by investigating the electrostatic and hydrogen bonding interactions between Kang Fuxin, chitosan, and fucoidan MNs (KCFMN). KCFMN has good biocompatibility and antibacterial ability and can penetrate the stratum corneum of rat skin, promote the penetration of KFX, overcome the problem of low transdermal penetration efficiency of traditional drugs, and promote full-layer wound healing in rats by reshaping epithelial tissue. Its healing-promoting activity was significantly greater than that of the KFX group that was directly treated. Therefore, MNs, as a formulation method, can effectively improve the efficiency of healing and are good carriers for promoting the healing of drug formulations, with great application prospects.

### 4.3. Electrospinning Nanofibers

Electrospinning is a common method for preparing nanofiber membranes. The prepared nanofibers have the advantages of a larger surface area-to-volume ratio, smaller pore size, and higher porosity, which allows for better absorption of exudate, improves wound penetration, and prevents infection [217]. It can also adapt to different wounds through customized structures and directly disperse drugs into various polymers to form ultrafine nanofiber structures, which can act as temporary barriers instead of damaging the skin. It can respond well to various complex diabetes wounds. Moreover, it has good biodegradability and is an ideal way to prepare wound scaffolds [218]. Its low cost and high formulation efficiency can meet the needs of industrial production [219].

Larijani et al. [220] developed an electrospinning collagen scaffold using collagen, which could be used to improve the physical and biological properties of wounds. The prepared formulation had good cell compatibility and superior mechanical properties, biological properties, and wound-healing ability. In the process of wound healing in diabetic rats, it significantly improved the tissue structure of the wounds and the wound healing rate.

At present, few studies have used electrospinning technology to prepare animal proteins or peptides into nanofiber membranes for wound healing, but it has been proven that this technology can be used for the formulation of protein and peptide formulations. Moreover, its customizability, biodegradability, and skin barrier similarity make it a prominent advantage in treating various types of difficult-to-heal and chronic wounds. Therefore, electrospinning nanofiber membranes is an ideal platform and a promising wound dressing for wound-healing applications [221].

### 4.4. Others 

In addition to existing reports on the preparation of animal proteins and peptides into various formulations, there are other formulations and dressings that have great potential in wound healing and have attracted attention. These formulations have their own advantages and features, which are summarized in Table 4.

In recent years, various formulations and dressings have been continuously updated, and various intelligent nano formulation systems have also been widely reported. An ideal wound dressing should have good biocompatibility, protect the wound bed, maintain wound hydration, allow for gas exchange with the environment, remove excess exudate, physically protect against microorganisms, and possess specific mechanical properties such as flexibility [222]. According to this standard for evaluation, various formulations and dressings have their own advantages and features. As a means for bioactive components to better exert therapeutic effects, formulations and dressings can be selected according to the physical and chemical properties of the bioactive components and the characteristics of the target disease so that the formulation can better serve the efficacy of the drug.

**Table 4:** Comparison of the performance of different formulations and dressings.

Formulation or Dressing	Biocompatibility	Improve Penetration	Allow for Gas Exchange	Against Microorganisms	Control Drug Release	Targeted Drug Delivery	Others	Ref.
hydrogel	+	+	+	+	+	+		[189,190,191]
microneedles			+	+	+	+	Bypass the stratum corneum and enter the epidermis/dermis layer Natural mechanical stimulation induces collagen deposition and recombination Change the local stress environment Anti scar treatment	[201,202,203,204,205]
electrospinning nanofibers	+	+	+	+			Customized structure to adapt to different wounds Replace damaged skin as a temporary barrier	[218]
nanoparticles					+	+	Packaged in other dressings to make formulations Some types of nanoparticles have antibacterial properties	[223,224,225,226]
microparticles	+	+		+			Packaged in other dressings to make formulations	[227,228,229,230]
membranes			+	+			Easy to use Allow for inspection of the wound bed without removing the dressing	[231,232,233]
silk fibroin	+	+	+	+			Customized into complex structures to ensure specific requirements Regulate cell proliferation and migration during inflammation	[234,235,236]
sponges			+				Multi-porous material support that can restore its shape after mechanical compression or stretching	[237]
foam		+	+				Fix the dressing on the affected area Painless removal	[232,238,239]

## 5. Discussions

Skin injury, as a common disease, especially the persistent skin damage caused by various chronic diseases, seriously affects people’s quality of life and even endangers human safety, and it has received widespread attention. In summary, existing research on protein bioactive compounds has mostly focused on bioactive parts, with limited research on individual components. Owing to the complex composition of the active part, there may be side effects and adverse reactions caused by heteroproteins, heteropeptides, or other unknown components, which pose significant challenges for subsequent formulation research. In subsequent formulation research, selecting appropriate indicators for characterization and evaluation of the formulation is difficult, and ensuring the uniformity and stability of the formulation quality is also impossible. In this literature review, we found that there was indeed limited research on this topic. Moreover, in clinical applications, because its impurity components may cause side effects and adverse reactions, it also presents great challenges for quality control, consistency between batches, and the standardization of production, which affects its clinical application. Therefore, follow-up studies should focus on monomer composition as much as possible and develop it into safe, economical, effective, and convenient modern formulations for clinical application. However, considering the low efficiency of extraction, separation, and purification in the study of protein components, it is difficult to clarify the composition of protein extracts. Moreover, protein synthesis is difficult and costly. Obtaining a single protein becomes a challenge. To solve these problems, it may be necessary to improve protein separation and purification or synthesis technologies. Moreover, the inherent instability of proteins also requires the use of formulation methods and special administration methods to deliver drugs to the target site while preventing protein denaturation. Of course, this issue is also one of the difficulties and key points that all protein drug research institutes must face.

Although it is easier to obtain a single component from peptides than from proteins, there are still some obstacles that need to be faced when applying peptides in clinical practice, such as toxicity and stability issues. The toxicity of peptides can be mainly divided into three categories: cytotoxicity (general), hemolytic (toxic to red blood cells), and immunotoxicity (regulating immune responses in an undesirable way) [240]. At the same time, peptide components also face the unstable properties that protein components need to face, such as short half-life, limited oral availability, and susceptibility to plasma degradation [241]. In response to these issues, in addition to conventional methods such as testing toxicity, formulation methods, or adjusting administration methods to improve stability, computer-aided drug design and artificial intelligence can also be used for early prediction to minimize risks during the screening stage of bioactive peptides. With the continuous development of computers and artificial intelligence, computing tools have changed pharmaceuticals at an astonishing speed [242,243]. There have emerged some models and algorithms that can be used to predict peptide toxicity, and peptides with ideal properties can be designed through model building methods, which have great potential in peptide science and computer-aided drug design. These methods have great practicality in accelerating the discovery and application of peptide drugs [244,245].

In addition, during the review process, it was found that some peptides not only promote wound healing but also have antibacterial effects, commonly found in extracts of amphibians. There have also been detailed studies reporting on how to regulate the wound microbiota through antibacterial forms, improve the microenvironment, and thereby affect the secretion of cytokines, adjusting the wound-healing process [56]. This type of dual-acting peptide has unique advantages in the field of wound healing, as it can promote wound healing while being antibacterial, effectively resist microbial invasion, and prevent repeated infections. This multifunctional peptide is attractive due to its unique biological activity and has made some progress [246]. In future research, particular attention can also be paid to multifunctional peptides.

The current research on the mechanism of action is relatively limited and often focuses on a certain signaling pathway. There has been little comprehensive research on the upstream- and downstream-related pathways and their underlying mechanisms of action. However, wound healing is a complex process involving cell proliferation, migration, matrix synthesis, and contraction, which requires the participation of multiple types of cells and pathways. The promotion of wound healing should also be the result of synergistic effects of multiple cells and pathways. Therefore, in addition to focusing on a single pathway, systematic research should be conducted to illustrate the entire process of the component’s action in vivo, guiding more scientific clinical medication.

Finally, topical administration is a common method for treating wounds. Topical administration can greatly avoid the problem of protein and peptide degradation, but it also faces issues such as short wound retention rate. But the continuous innovation of formulation methods and dressings provide more selectivity and possibility for the carrier of wound healing. When choosing the formulation method, it is not only limited to the existing common formulation methods, but suitable formulations can also be selected based on the physical and chemical properties of the active substance and the characteristics of the disease to better serve the drug and further improve its healing effect. In addition, it is also possible to co-load antibacterial ingredients through formulation methods to improve the phenomenon of delayed wound healing caused by bacterial infection in chronic wounds and further improve the efficiency of healing to help humanity overcome the difficult problem of wound healing as soon as possible.

## Figures and Tables

**Table 1:** Summary of proteins and peptides from animal sources that promote wound healing.

Source	Species	Active Ingredient	Acquisition Method	Effect on Wound Healing	Ref.
Earthworm	*Eisenia foetida*	G-90	Remove impurities with chloroform Extract with methanol and water	Promote EGF generation Promote proliferation of fibroblasts and epithelial cells	[23,24]
	Ohira the 2nd	EE	Ultrafiltration	Stimulate cytokine secretion Anti-inflammatory Increase the synthesis of collagen Promote blood capillary Promote fibroblast proliferation	[10]
	Ohira the 2nd	EE-1	Ultrafiltration, dialysis	Activate angiogenesis Activate epithelial regeneration Inhibite fibrosis Inhibite of scar formation	[25]
	*Eisenia foetida*	ES-2	Homogenize, dialysis, salting out	Stimulate VEGF production Promote proliferation of fibroblasts and keratinocytes	[26]
	*Eisenia foetida*	G-90′	Homogenize, dialysis, salting out	Upregulate expression of proteins HSP70 and lysozyme	[27]
	*Eisenia Andrei*	col4a1	Sonication, centrifuge, filter	Activate the RAS/MAPK signaling pathway Activate the proliferation of fibroblasts	[13]
	*Pheretima vulgaris* Chen	PvE-3	Extracted by water Precipitated by ethanol	Anti-inflammatory Stimulate granulation formation Enhance the secretion of α-SMA Promote the neovascularization and collagen fiber synthesis	[28]
	*Eisenia foetida*	ES2	Dialysis, centrifuge	Promote the secretion of cytokines Promote the synthesis and accumulation of collagen	[29]
	earthworm	EAP	——	Accelerate the proliferation of NIH3T3 Promote the expression of cyclins	[30]
Periplaneta	*Periplaneta americana* L.	PAL	Ethanol extraction	Regulate cell cycle Promote cytokine secretion Accelerate epithelial cell growth Promote angiogenesis	[31]
	*Periplaneta americana*	PaPPc2 and PaPPc3	Reflux extracted by water Precipitated by ethanol Anion exchange chromatography	Promote angiogenesis Promote collagen synthesis	[32]
	*Periplaneta americana*	PAE	Ethanol extraction, filter	Enhance epithelial repair, follicle regeneration, and fibrous tissue proliferation	[19]
Amphibians	*Amolops jingdongensis*	*Bv8-AJ*	Ladder sequencing determines amino acid sequence Synthetic peptides	Anti-inflammatory Promote IL-1 generation Promote proliferation of fibroblasts and keratinocytes	[33]
	*Duttaphrynus melanostictus*	cathelicidin-DM	cDNAs synthesis for determining amino acid Sequences of synthetic peptides	Facilitate the proliferation of human umbilical vein endothelial cells (HUVECs), human skin fibroblasts (HSFs), and human immortalized epidermal cells (HaCaTs) Promote the migration of HUVEC and HSF cells Promote the differentiation of fibroblasts into myofibroblasts	[34]
	*Hyla annectans*	FW-1 and FW-2	cDNAs synthesis for determining Amino Acid	Anti-inflammatory Antioxidant	[35]
	*Nanorana ventripunctata*	Cathelicidin-NV	Ladder sequencing determines amino acid sequence Synthetic peptides	Promote the expression of cytokine Enhance the proliferation of keratinocyte and fibroblast cells Promte the differentiation of fibroblasts to myofibroblasts Promote collagen production	[36]
	*Nanorana ventripunctata*	Antioxidin-NV	Ladder sequencing and cDNAs synthesis determines amino acid sequence Synthetic peptides	Anti-inflammatory Antioxidant Alleviate DNA damage Alleviate cell apoptosis	[37]
	*Odorrana andersonii*	OA-GL21	Ladder sequencing and cDNAs synthesis determines amino acid sequence Synthetic peptides	Induce the migration of HaCaT and HSF cells Promote re-epithelialization Promote formation of granulation tissue	[38]
	*Odorrana andersonii*	OA-GL12	cDNA synthesis, Illumina MiSeq sequencing, Synthetic peptides	Promote the expression of cytokine Anti-inflammatory Antioxidant	[39]
	*Odorrana andersonii*	OA-GL17d	DNA cloning, mass spectrometry and Edman degradation determines amino acid sequence. Synthetic peptides	Promote the expression of TGF-β1	[40]
	*Odorrana grahami*	AH90	Solid-phase synthesis	Induce the release of TGF-β1	[41]
	*Odorrana grahami*	CW49	Solid-phase synthesis	Anti-inflammatory Promote angiogenesis	[42]
	*Odorrana margaretae*	OM-LV20	Edman degradation and cDNA cloning determines amino acid sequence. Synthetic peptides	Induce the proliferation of HaCaTs	[43]
	*Odorrana andersonii*	Cathelicidin-OA1	Ladder sequencing and cDNAs synthesis determines amino acid sequence Synthetic peptides	Enhance the recruitment of macrophages Accelerating re-epithelialization Enhance granulation tissue formation	[44]
	*Odorrana tormota.*	Ot-WHP	Synthetic peptides	Elicite the production of regulatory factors Initiate and accelerate the inflammatory phase Promote keratinocyte migration	[45]
	*Pelophylax* kl. *Esculentus*	Brevinin-2Ta	DNA cloning, and mass spectrometry determines amino acid sequence Solid-phase synthesis	Anti-inflammatory Promote epithelial migration Promote angiogenesis	[46]
	*Pelophylax nigromaculatus*	Brevinin-2PN	cDNA sequencing determines amino acid sequence.	Accelerate the healing of HSF scratches Enhance cell migration Stimulate gene expression of growth factors	[47]
	*Polypedates megacephalus*	PM-7	Ladder sequencing determines amino acid sequence	Enhance proliferation and migration in HUVECs and HSFs	[48]
	*Rana limnocharis*	RL-RL10	——	Promote proliferation and migration of keratinocytes	[49]
	*Rana limnocharis*	RL-QN15	Ladder sequencing determines amino acid sequence Synthetic peptides	Modulate the secretion of cytokines Regulate the generation of TGF-β1 and TGF-β3 Accelerated re-epithelialization and granulation tissue formation	[50]
	*Tylototriton verrucosus*	Tylotoin	Ladder sequencing and cDNA synthesis determines amino acid sequence Synthetic peptides	Promote the release of TGF-β1 and IL-6 Enhance the motility and proliferation of keratinocytes vascular endothelial cells, and fibroblasts Accelerate re-epithelialization and granulation tissue formation	[51]
	salamander *Tylototriton kweichowensis*	TK-CATH	cDNAs synthesis determines amino acid sequence Synthetic peptides	Anti-inflammatory Promote the expression of cytokines Promote the motility and proliferation of keratinocytes	[52]
Marine organisms	*Oreochromis niloticus*	TP2-5 and TP2-6	Solid-phase synthesis	Promote the motility and proliferation of cells Promote neovascularization Promote the release of cytokines	[18]
	Sea cucumber	VTPY and VLLY	Synthetic peptides	Promote the proliferation and migration of HSFs and HUVECs Increase mitochondrial respiratory capacity Block the binding of MKP to ERK2 and PHLPP to AKT	[53]
	sea cucumber (*S. japonicus*)	SCP	Alkali soluble acid precipitation method Anion ion exchange chromatography	Boost epithelialization, dermis integrity, vascularization, and collagen deposition Adjust the immune cells	[54]
	*Sipunculus* *nudus*	SNCP	Hydrolyzed with acetic acid solution with pepsin	Reduce inflammation Improve collagen deposition and recombination Blockade of the TGF-β/Smads signaling pathway	[3]
	*Salmon salar*	Ss-SCP	Hydrolyzed with collagenase and compound protease	Upregulate wound NOD2 and BD14 Implicate in microflora colonization regulation Control inflammatory reaction Increase wound angiogenesis and collagen deposition	[55]
	*Tilapia nilotica*	Tn-SCP	——	Upregulate wound NOD2 and BD14 Implicate in microflora colonization regulation Control inflammatory reaction Increase wound angiogenesis and collagen deposition	[55]
	*Nibea japonica*	MCPs	Hydrolyzed with neutral protease	Increase the protein levels of NF-κB p65, IKKα and IKKβ Increase the expression of EGF, FGF, VEGF, and TGF-β	[56]
	*Theragra chalcogramma*	PCP	Enzymolysis, ultrafiltration	Promote the expression of cytokines Increase wound healing rate, hydroxylproline content, collagen deposition and tensile strength	[57]
	*Theragra chalcogramma*	FPP	Hydrolyzed with trypsin and alkaline protease	Promote the expression of cytokines Increase wound healing rate, hydroxylproline content, collagen deposition and tensile strength	[57]
Scorpions	Scorpion	SVAP	——	Anti-inflammatory Anti-infective	[58]
	*Tityus stigmurus.*	Stigmurin	Synthetic peptides	Antioxidant Antibacterial	[59]

**Table 2:** Wound-healing peptides relevant for amphibian skin defense.

Peptide	AA Sequences	Length (AA)	N-Modification	C-Modification	Disulfide Bond	*Species*	Ref.
Bv8-AJ	AVITGACERDVQCGGGTCCAVSLWMQGLRICTPLGRQGENCHPASRKVPFAGLRLHNSCPCQSNLACKTLPNGKYQCMPS	81			+	*Amolops jingdongensis*	[33]
Bombesin	QRLGNQWAVGHLM	13	Pyr			*Bombina bombina* *Bombina variegata* *Bombina orientalis*	[99]
Cathelicidin-DM	SSRRKPCKGWLCKLKLRGGYTLIGSATNLNRPTYVRA	37			+	*Duttaphrynus melanostictus*	[34,100]
FW-1	FWPLI	5		NH_2_		*Hyla annectans*	[35]
FW-2	FWPMI	5		NH_2_		*Hyla annectans*	[35]
Cathelicidin-NV	ARGKKECKDDRCRLLMKRGSFSYV	24			+	*Nanorana ventripunctata*	[36]
Antioxidin-NV	GWANTLKNVAGGLCKMTGAA	21				*Nanorana ventripunctata*	[37]
OA-GL21d	GLLSGHYGRVVSTQSGHYGRG	21				*Odorrana andersonii*	[38]
OA-GL12	GLLSGINAEWPC	12				*Odorrana andersonii*	[39]
OA-GL17	GLFKWHPRCGEEQSMWT	17				*Odorrana andersonii*	[40]
AH-90	ATAWDFGPHGLLPIRPIRIRPLCG	25				*Odorrana grahami*	[41]
CW49	APFRMGICTTN	11				*Odorrana grahami*	[42]
Cathelicidin-OA1	IGRDPTWSHLAASCLKCIFDDLPKTHN	27				*Odorrana margaretae*	[44]
Ot-WHP	ATAWDLGPHGIRPLRPIRIRPLCG	24				*Odorrana tormota*	[45]
Brevinin-2Ta	GILDTLKNLAKTAGKGILKSLVNTASCKLSGQC	33			+	*Pelophylax* kl. *Esculentus*	[46]
Brevinin-2PN	GLMDSLKGLAATAGKTVLQGLLKTASCKLEKTC	33			+	*Pelophylax nigromaculatus*	[47]
PM-7	FLNWRRILFLKVVR	14				*Polypedates megacephalus*	[48]
RL-RL10	RLFKCWKKDS	10				*Rana limnocharis*	[49]
RL-QN15	QNSYADLWCQFHYMC	15			+	*Rana limnocharis*	[50]
Tylotoin	KCVRQNNKRVCK	14			+	*Tylototriton verrucosus*	[51]
TK-CATH	GGQDTGKEGETGKKKKSDNWFMNLLNKFLELIGLKEAGDD SEPFCFTCIFDMFSQ	55				*Tylototriton* *kweichowensis*	[52]

**Table 3:** Pathways by which animal protein and peptide components promote wound healing.

Signaling Pathway	Components
PI3K/AKT	EAP [30], ES2 [29], col4a1 [13], PvESII [28], Tylotoin [51], TK-CATH [52], AH-90 [41], Ot-WHP [45], Bv8-AJ [33], RL-QN15 [50], VTPY and VLLY [53], SCP [54]
TGF-Beta	AH-90 [41], SNCP [3], Ot-WHP [45], RL-QN15 [50], PAE [19], Antioxidin-NV [37], OA-GL17d [80]
NF-κB	AH-90 [41], Ot-WHP [45], FW-1 [35], FW-2 [35], MCPs [56]
JAK/STAT	PAE [19]
